# Draft Genome Sequence of Antagonistic Agent *Lysobacter antibioticus* 13-6

**DOI:** 10.1128/genomeA.00566-14

**Published:** 2014-10-09

**Authors:** Lihong Zhou, Miao Li, Jun Yang, Lanfang Wei, Guanghai Ji

**Affiliations:** a Key Laboratory of Agricultural Biodiversity for Plant Disease Management under the Ministry of Education, Yunnan Agricultural University, Kunming, People’s Republic of China; b Experimental Agricultural Center, Yunnan Agricultural University, Kunming, People’s Republic of China

## Abstract

*Lysobacter antibioticus* 13-6, isolated from the roots of Chinese cabbage, effectively controls the pathogens *Plasmodiophora brassicae, Xanthomonas oryzae* pv. oryzicola, *X. oryzae* pv. oryzae, *Xanthomonas axonopodis* pv. dieffenbachiae, and *Pseudomonas syringae* pv. tabaci. We report the first draft genome sequence of the *L. antibioticus* species in China.

## GENOME ANNOUNCEMENT

The genus *Lysobacter* represents a source of biocontrol agents, which consisted of 16 species (1, 2). Recently, we reported that application of *L. antibioticus* 13-6 can control *brassicae* clubroot (3). Due to its tremendous potential application value, we analyzed the draft genome sequence of *L. antibioticus* 13-6.

13-6 genomic DNA was extracted from cell grown at 28°C in Luria-Bertani agar (LB) liquid cultures by using a genomic-tip 100/G kit (Qiagen GmbH, Hilden, Germany). DNA shotguns libraries were constructed using 1-kb fragments which were break-interrupted by Fisher. A TruSeqTM DNA sample prep kit—Set A (Illumina, USA), TruSeq PE cluster kit, and Illumina Hiseq2500 were used for the construction of libraries, amplification, and sequencing, respectively. A total of 11,524,134 filtered reads for the 13-6 genome were assembled into 162 contings (*N*_50_ length, 60,432), with an average coverage of 40.0×, using the A5 pipeline. The genome has a G+C content of 67.14%, which is similar to the content of the strain of *L. capsici* AZ78 (4); 4,056 genes belong to homologous sequences in the NCBI nr database, in which 2,674 genes were assigned functions by using the NCBI Prokaryotic Genomes Annotation Pipeline utilizing Gene-MarkS.

The analysis showed that the genome contains genes involved in phenylalanine, lipoic acid, laurine, and hypotaurine metabolism. Betalain synthetic genes were also found in the 13-6 genome. As excepted, the 13-6 genome contains a high number of genes coding for antibiotics, a property that explains its resistance to plant pathogens, specifically, tetracycline, vancomycin, β-lactam, streptomycin, novobiocin, penicillin, cephalosporin, butirosin, and neomycin.

The availability of the draft genome of *L. antibioticus* 13-6 will help elucidate the mechanism of disease prevention and will provide background knowledge for its production and application.

### Nucleotide sequence accession number.

This whole-genome shotgun project has been deposited at GenBank under the accession no. JMTZ00000000.
